# Large variability in minimal clinically important difference, substantial clinical benefit and patient acceptable symptom state values among literature investigating patellar stabilization surgery: A systematic review

**DOI:** 10.1002/ksa.12684

**Published:** 2025-05-06

**Authors:** Ahmed Bilgasem, Prushoth Vivekanantha, Lauren Gyemi, Zackariyah Hassan, David Slawaska‐Eng, Amit Meena, Shahbaz Malik, Darren de SA

**Affiliations:** ^1^ Northern Ontario School of Medicine Sudbury Ontario Canada; ^2^ Division of Orthopaedic Surgery, Department of Surgery McMaster University Hamilton Ontario Canada; ^3^ Michael DeGroote School of Medicine McMaster University Hamilton Ontario Canada; ^4^ Department of Orthopaedics and Trauma Shalby Hospital Jaipur Jaipur India; ^5^ Department of Orthopaedic Surgery Worcestershire Acute Hospitals NHS Trust Worcester UK

**Keywords:** medial patellofemoral ligament reconstruction, minimal clinically important difference, patellar instability, patient acceptable symptom state, substantial clinical benefit

## Abstract

**Purpose:**

To investigate minimal clinically important difference (MCID), substantial clinical benefit (SCB), patient acceptable symptom state (PASS) values for patient‐reported outcome measures (PROMs) after patellar stabilization surgery for patellar instability. Secondary outcomes included to describe methods to calculate clinically significant outcomes (CSOs), and to report on the achievement of these metrics.

**Methods:**

On 31 July 2024, three databases were searched. Information on whether studies calculated MCID, SCB or PASS values or used previously established values was recorded. Data on study characteristics, CSO values, and the method of MCID quantification (e.g., distribution vs. anchor) were extracted.

**Results:**

A total of 17 articles with 1447 patients (1462 knees) were included. A total of 18 unique outcome measures were reported. Six out of 15 (40%), 2 out of 5 (40%), and zero studies used prior established values for MCID, SCB and PASS, respectively. MCID ranged widely (e.g., International Knee Documentation Committee [IKDC]: 5.6–20.5; Kujala Anterior Knee Pain Scale: 5.38–11.9 and Lysholm: 5.6–11.1). Fourteen out of 15 utilized a distribution‐based method to calculate MCID, with only one study using an anchor‐based method. SCB values ranged widely as well (e.g., IKDC: 14.5–23.6; Knee Osteoarthritis and Outcome Score [KOOS] symptoms: 4.2–14.2 and KOOS activities of daily living [ADLs]: 6.5–25.7). Large variability was found among percentages of patients that achieved MCID values (e.g., IKDC: 28%–98.6%, Kujala: 38%–100%, Lysholm: 44%–98.4% and Tegner: 84%–95%).

**Conclusion:**

The significant heterogeneity in reported thresholds for MCID, SCB and PASS across studies highlights critical challenges in interpreting results after patellar stabilization surgery, specifically regarding what constitutes a clinically relevant outcome. MCID was the most commonly reported metric and calculated predominantly with distribution‐based methods, with over half of the studies using previously established thresholds. PASS and SCB were widely underreported as well, suggesting a need for studies investigating patellar stabilization to prioritize the calculation of all three metrics, using anchor‐based techniques.

**Level of Evidence:**

Level IV.

**Abbreviations**
ADL: activities of daily living
BPI: Banff patellofemoral instability instrument
CSO: clinically significant outcome
IKDC: international knee documentation committee
JR: joint replacement
KOOS: knee osteoarthritis and outcome score
MCID: minimal clinically important difference
MPFL: medial patellofemoral ligament reconstruction
PASS: patient acceptable symptom state
PRISMA: Preferred Reporting Items for Systematic Review and Meta‐Analyses
R‐AMSTAR: Revised Assessment of Multiple Systematic Reviews
SCB: substantial clinical benefit

## INTRODUCTION

Patellar dislocation and instability are common orthopaedic injuries that can significantly affect a patients' quality of life, functionality, and can result in long‐term consequences including recurrent dislocations, anterior knee pain and ultimately patellofemoral arthritis [10, 46]. The difficulties of managing these conditions underscore the necessity for quantifiable measures of treatment efficacy. Recently, orthopaedic literature has begun to evaluate treatment efficacy through the use of psychometric measures of clinical significance [14]. Understanding psychometric clinically significant outcomes (CSOs)—such as the minimal clinically important difference (MCID), patient acceptable symptom state (PASS) and substantial clinical benefit (SCB)—allows clinicians and researchers to better capture patient‐perceived outcome change that has a meaningful impact on their health‐related quality of life [17, 20, 26, 49].

Specifically, the MCID represents the smallest change in a patient‐reported outcome measure (PROM) that patients perceive as beneficial, while the PASS defines the threshold at which patients consider their symptoms acceptable [5, 17, 45]. SCB, on the other hand, encompasses a broader perspective, representing the amount of change needed for patient‐perceived significant improvement that enhances overall patient well‐being [31]. These values are commonly calculated for PROMS in orthopaedics to determine the clinical effectiveness of the surgical intervention and provide evidence to measure decreases in pain and improvements in function or quality of life [1]. Examples of PROMs used for knee surgeries include the Knee Injury and Osteoarthritis Outcome Score (KOOS), International Knee Documentation Committee (IKDC) Score, and Kujala Anterior Knee Pain Score [15, 18, 37]. The MCID can be calculated through distribution‐based or anchor‐based methods, while the PASS and SCB are commonly calculated through anchor‐based methods. Both methods can be further subdivided based on their analysis; distribution methods utilize standard deviations and standard error measurements, and anchor methods determine a baseline ‘anchor’ for comparison of improvement through mean differences or receiver operating curves (ROCs) [40]. The benefits of distribution‐based methods are their quick ability to draw on sample data to estimate MCID, however they fail to consider patient's subjective feelings of improvement which are captured by anchor‐based methods [40]. While both methods have been reported to show heterogeneity in MCID calculations [5, 45], there are arguments in favour of anchor‐based methods largely due to their ability to incorporate patient's feelings of improvement [8, 40]. These concepts are particularly relevant in the context of patellar instability, where treatment goals extend beyond anatomical correction to include functional recovery, return to sport, and overall patient satisfaction [25, 42].

Recent literature has begun to establish MCID, SCB and PASS values for various PROMs specific to patellar instability [13]. However, discrepancies and heterogeneity exist, largely due to differences in methodologies and patient populations. Therefore, the aim of this systematic review is to summarize existing literature on MCID, SCB and PASS in patients with patellar instability undergoing patellar stabilization procedures such as medial patellofemoral ligament reconstruction (MPFLR) or tibial tubercle osteotomy (TTO), in terms of thresholds, calculation methods and percentages of achievement. It is hypothesized that there will be significant heterogeneity across established thresholds and in terms of methods of calculation.

## MATERIALS AND METHODS

During the development of this manuscript, the Preferred Reporting Items for Systematic Reviews and Meta‐Analyses (PRISMA) and Revised Assessment of Multiple Systematic Reviews (R‐AMSTAR) guidelines for coordinating and reporting systematic reviews were followed [19, 23].

### Search strategy and screening

On 31 July 2024, three online databases (MEDLINE, EMBASE and PubMed) were searched to identify literature outlining MCID, SCB or PASS values for patients who underwent treatment for patellar instability. The search strategy combined terms such as ‘patellar dislocation’, ‘patellar instability’, ‘medial patellofemoral ligament’, ‘tibial tubercle osteotomy’, ‘minimally clinically important difference’, ‘MCID’, ‘patient acceptable symptom state’, ‘PASS’, ‘substantial clinical benefit’ and ‘SCB’ (Supporting Information S1: Table 1). This was supplemented by a manual search of references from included studies to ensure all relevant articles were included.

Study screening was conducted on Covidence (Veritas Health Innovation). Studies that met the following criteria were included in this review: (1) studies including patients with first‐time or recurrent patellar dislocations treated surgically or non‐surgically, (2) studies that outlined a MCID, PASS or SCB value either newly calculated from that specific sample or taken from a previous study, (3) human patients and (4) studies published in the English language. Exclusion criteria included (1) cadaveric or biomechanical studies, (2) systematic reviews or meta‐analyses, (3) review articles, (4) case reports or studies with less than five patients to ensure sample size and data robustness, (5) conference abstracts and (6) textbook chapters.

### Screening

Title and abstract screening, as well as full‐text screening, were done by two authors (AB and ZH) with any conflicts resolved among them. A senior author (PV) was consulted to review conflicts that could not be resolved. During full‐text screening, studies were independently screened by two authors, and disagreements were resolved in similar fashion.

### Assessment of agreement

The inter‐reviewer agreement was evaluated using the kappa (*κ*) statistic for screening. A priori classification was defined according to the following criteria: *κ* of 0.91–0.99 was considered almost perfect agreement; *κ* of 0.71–0.90 was considerable agreement; *k* of 0.61–0.70 was high agreement; *κ* of 0.41–0.60 was moderate agreement; *κ* of 0.21–0.40 was fair agreement and a *κ* or ICC value of 0.20 or less was classified as no agreement [30].

### Quality assessment

Quality assessment of included studies was completed using the Methodological Index for Non‐Randomized Studies (MINORS) [44]. According to these criteria, non‐comparative studies could get a maximum score of 16 and comparative studies could get a maximum score of 24 [44]. Based on a previous systematic review, the classification for non‐comparative studies was as follows: 0–4 indicated very low quality evidence, 5–7 indicated low quality evidence, 8–12 indicated fair quality evidence and scores ≥13 indicated high quality evidence [47]. Comparative studies were categorized as: 0–6 very low quality, 7–10 low quality, 11–15 fair quality, 16–20 good quality and ≥20 high quality [47].

### Data abstraction

Two authors performed independent data abstraction on the included articles. Study characteristics and demographics included title, study design, number of patients and knees, gender, mean age and mean follow‐up. For MCID, PASS and SCB, values were recorded regardless of whether they were newly calculated or taken from a previous study. If not calculated, the specific reference study was also extracted. Quantification methods (e.g., distribution or anchor) were also recorded. Furthermore, the percentage of patients who achieved the MCID, PASS and SCB was recorded.

### Statistical analysis

Results were presented in a descriptive summary format. Means, ranges, percentages, and standard deviations (SDs) were calculated using Google Sheets (Google LLC). If a study analyzed associated statistical parameters, *p* values were recorded.

## RESULTS

### Literature search and study quality

**Figure 1:**
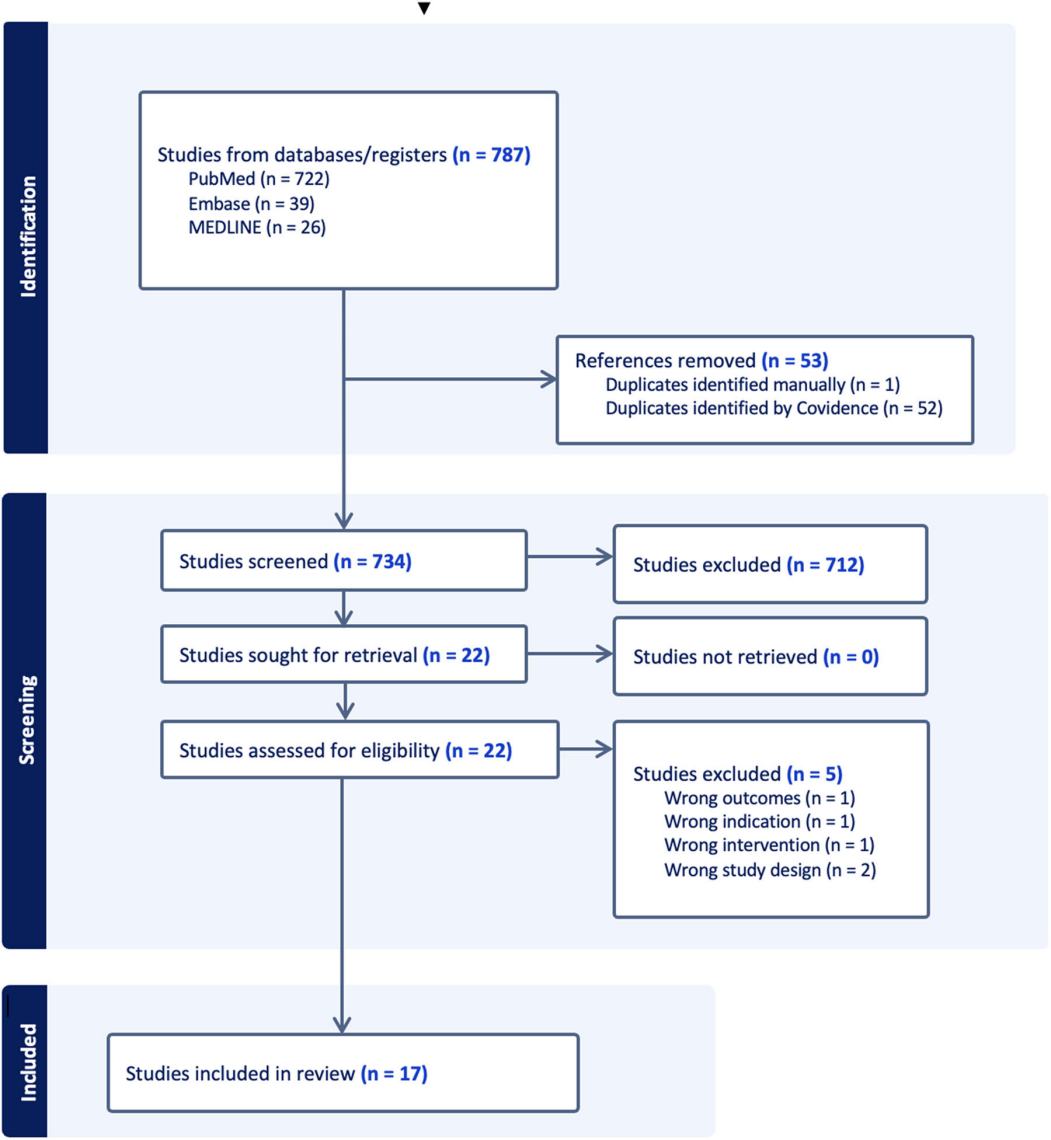
Preferred Reporting Items for Systematic Reviews and Meta‐analyses flow diagram representing a systematic review assessing clinically significant outcome measures for patients with patellar instability.

The initial search of databases produced 787 articles, with 53 articles removed as duplicates. A total of 734 titles and abstracts were screened, with 712 identified as irrelevant. Twenty‐two full texts were assessed for eligibility, and overall 17 satisfied the previously mentioned inclusion criteria (Figure 1). The agreement between reviewers was considerable during the title and abstract screening and full‐text screening stages, with kappa values of 0.875 (95% confidence interval [CI]: 0.766–0.983) and 0.861 (95% CI: 0.597–1.000), respectively. The articles in this review included 12 retrospective cohort studies (Level III evidence), 1 retrospective case–control (Level III evidence) and 4 case series (Level IV evidence) [2, 7, 14, 24, 28, 32, 34, 35, 36, 39, 41, 43, 50, 51, 55, 56, 57]. The mean MINORS score among comparative and non‐comparative studies was 10.8 and 15.5, respectively, or of fair quality (Table 1).

**Table 1:** Demographics.

Author	Study design	Number of patients/knees	Female (%)	Mean age (SD)	Mean follow‐up ± SD (months)	Lost to follow‐up (%)	Surgical procedure	MINORS (/16,*/24*)
Bayoumi (2021)	Case series (IV)	32/32	24 (75%)	Median (IQR): 21.0 (19.0–25.0)	22.0 (4.4)	0	TTO (all)	9
Hu (2024)	Retrospective cohort (IV)	128/133	86 (65%)	22.3 (6.6)	50.6 (22.1)	0	MPFLR (*n* = 72) MPFLR + MQTFLR (*n* = 61)	10
Long (2023)	Case series (IV)	61/63	42 (69%)	22.0 (7.2)	42.0 Range: 12–80.4	15 (24.6) at 12 months 27 (44.3) lost for PROM data	MPFLR (all) TTO (*n* = 16)	10
Mao (2024)	Case series (IV)	38/38	27 (71%)	16.1 Range: 14.1–17.8	67.2 Range: 4.0–7.6 years	0	MPFLR + TTO (all)	11
Milinkovic (2023)	Retrospective cohort (III)	57/57	39 (68%)	26.5 (6.3)	32.1 (7.1) Min. 24 months	NR	MPFLR (all) TTO: *n* = 32 Trochleoplasty: *n* = 6 DFO: *n* = 18	*18*
Milinkovic (2024)	Retrospective cohort (III)	237/237	166 (70%)	22.4 (6.8)	34.9 (13.4)	NR	MPFLR (*n* = 210) TTO: *n* = 74 Trochleoplasty: *n* = 74 Varisation/rotational DFO (*n* = 17)	11
Qiao (2024)	Retrospective cohort (III)	142/142	99 (69.7%)	23 (7.0)	Median: 58 (20–88) Min. 18 months	0	MPFLR (all) ± TTO (not specified)	11
Qiao (2024)	Retrospective cohort (III)	36/36	31 (86%)	20.6 Range: 16–38 SD est.: 5.6	Median: 30 (21–39) Minimum 18 months	0	MPFLR (all) TTO (*n* = 18) DFO (*n* = 18)	*20*
Retzky (2024)	Retrospective cohort (II)	45/46	34 (76%)	Median (IQR): 25.3 (20.2–33.5)	Median 20.4 (13.2–24.0) Minimum 12 months	NR	TTO (all)	10
Runer (2024)	Retrospective case–control (III)	64/64	30 (46.875%)	20.0 (6.3)	28.7 (7.5)	0	MPFLR (all)	*18*
Sharma (2023)	Retrospective cohort (III)	97/97	49 (50.5%)	24.6 (9.3)	Minimum 12 months	0	MPFLR (all)	12
Shi (2023)	Case series (IV)	39/42	27 (69.2%)	22.2 (7.6)	47.3 (20.2)	0	MPFLR + MQTFLR (all)	12
Walsh (2022)	Retrospective cohort (III)	139/139	90 (65%)	21.7 (8.2)	Minimum 6 months	56 (40%) at 6 months 83 (60%) at 1 year	MPFLR (all)	12
Waters (2024)	Retrospective cohort (III)	49/49	34 (69.4%)	22.1 (10.8)	27.6 (9.8)	NR	MPFLR (all)	*15*
Zhang (2023)	Retrospective cohort (III)	54/54	13 (24.1%)	21.6 (5.0)	82.6 (15.9)	0	MPFLR (all) TTO (*n* = 18)	*20*
Zimmerman (2020)	Retrospective cohort(III)	75/75	48 (64%)	23.8 (5.0)	26.7 (12.1)	0	Revision MPFLR (*n* = 25) Primary MPFLR (*n* = 50) TTO (*n* = 15) Trochleoplasty (*n* = 18)	*19*
Zimmerman (2023)	Retrospective cohort study (III)	122/122	80 (65.6%)	22.2 (6.0)	37.9 (9.3)	NR	MPFLR (*n* = 38) TTO (*n* = 39) DFO (*n* = 7) Trochleoplasty (*n* = 67)	*17*

Abbreviations: DFO, distal femoral osteotomy; IQR, interquartile range; MINORS, Methodological Index for Non‐randomized Studies; MPFLR, medial patellofemoral ligament reconstruction; MQTFL, medial quadriceps tendon femoral ligament; NR, not reported; SD, standard deviation; TTO, tibial tubercle osteotomy.

### Study characteristics

A total of 17 articles were included, with 1447 patients and 1462 knees. The mean ages ranged from 16.1 to 26.5 years, with a pooled mean age of 23.0 years. Females comprised 70% of the total 1447 patients. The pooled mean follow‐up time was 34.2 months (range of means: 6–82.6 months). Two studies did not report mean or median follow‐up times and used minimum intervals at 6 and/or 12 months [41, 50]. Patients lost to follow‐up were reported in 12 studies and ranged from 0% to 60%. Two studies reported direct loss to follow‐up times from patients included in their analysis [24, 50]. Three studies described calculation of CSOs to be the primary outcome of the study [34, 36, 50] (Table 1).

### MCID

Fifteen studies (15/17; 88.2%) comprising 1335 patients reported MCID values for patients with patellar instability [2, 7, 14, 28, 32, 34, 35, 36, 41, 43, 50, 51, 55, 56, 57]. Among these studies, nine articles (9/15; 60%) calculated MCIDs for their subject PROM data [7, 14, 28, 34, 36, 43, 50, 51, 57], while six articles (6/15; 40%) used previously established MCID values [1, 21, 24, 34, 35, 36]. The method of calculating MCID was primarily achieved through distribution‐based methods (14/15; 93.3%), while one study (1/15; 6.7%) used both distribution and anchor‐based studies to improve predictive power [50].

A total of 18 different outcome measures had MCIDs calculated or used established numbers. Studies that reported outcomes with multiple domain subsection values were analyzed individually. One study used established MCID values from the same author who had already been captured by this review [35]. The IKDC subjective score had eight MCID values reported, where seven values (7/8; 87.5%) were calculated [14, 34, 36, 43, 50] and one value (1/8 12.5%) was previously established [41]. The IKDC subjective score had an MCID range of 5.6 to 20.5. The Kujala score had six MCID values reported, with four values (4/6; 66.7%) being calculated [14, 28, 34] and two (2/6; 33.3%) being previously established [2, 54]. The range for Kujala MCID values was 5.38–11.9. The Lysholm score had four MCID values, with three values (3/4; 75%) being calculated [14, 34] and one (1/4; 25%) being previously established [54]. The range for the Lysholm score was 5.6–11.1. The Tegner Activity score had four MCID values reported, all of which were calculated and ranged from 0.58 to 0.9 [14, 28, 34]. The KOOS domains of Pain, Symptoms, Activities of Daily Living (ADLs), Sports and Recreation and Quality of Life each had four MCID values, all of which were calculated [34, 36, 50]. The MCID ranges for KOOS Pain, Symptoms, and ADL domains are 4.2–10.1, 9.6–10.8 and 7.2–10.2, respectively. For KOOS Sports and Recreation and Quality of Life, the MCID ranges are 12.4–17.8 and 12.2–13.2, respectively. MCID values for KOOS, KOOS Joint Replacement (JR) Pain, KOOS JR Symptom, KOOS JR ADL, Kujala Function, Kujala Symptom, IKDC Function, BPI 2.0 and VAS scores can be seen in Table 2.

**Table 2:** Minimal clinically important difference values.

Patient‐reported outcome measure	Author(s)	Calculated or established	Minimal clinically important difference calculation	Minimal clinically important difference
IKDC score	Hu (2024)	Calculated	Distribution	5.6 8.2
	Qiao (2024)	Calculated	Distribution	9.9
	Qiao (2024)	Established	Distribution	9.9
	Retzky	Calculated	Distribution	11.2
	Shi (2023)	Calculated	Distribution	9.2
	Sharma (2023)	Established	Distribution	20.5
	Walsh (2022)	Calculated	Distribution	9.7 8.6
IKDC Function score	Waters	Calculated	Distribution	1.54 1.71
Kujala Anterior Knee Pain Score	Bayoumi (2021)	Established	Distribution	11.9
	Hu (2024)	Calculated	Distribution	5.5 7.6
	Mao (2024)	Calculated	Distribution	5.38
	Qiao (2024)	Calculated	Distribution	9.1
	Qiao (2024)	Established	Distribution	9.1
	Zhang (2023)	Established	Distribution	8
Kujala Function score	Walsh	Calculated	Anchor Distribution	8.5 9.9
Kujala Symptoms score	Walsh	Calculated	Distribution Anchor	9.8 13.5
Lysholm score	Hu (2024)	Calculated	Distribution	5.6 6.7
	Qiao (2024)	Calculated	Distribution	11.1
	Qiao (2024)	Established	Distribution	11.1
	Zhang (2023)	Established	Distribution	10
Tegner Activity score	Hu (2024)	Calculated	Distribution	0.7 0.8
	Mao (2024)	Calculated	Distribution	0.58
	Qiao (2024)	Calculated	Distribution	0.9
	Qiao (2024)	Established	Distribution	0.9
BPI 2.0 score	Milinkovic (2024)	Calculated	Distribution	9.5
	Zimmerman (2023)	Calculated	Distribution	9.9
	Milinkovic (2023) and Zimmerman (2020)	Established	Distribution	6.2
KOOS Total score	Bayoumi (2021)	Established	Distribution	10
KOOS Pain score	Qiao (2024)	Calculated	Distribution	9.0
	Qiao (2024)	Established	Distribution	9.0
	Retzky (2024)	Calculated	Distribution	10.1
	Walsh (2024)	Calculated	Distribution Anchor	4.2 8.3
KOOS Symptoms score	Qiao (2024)	Calculated	Distribution	10.8
	Retzky (2024)	Calculated	Distribution	10.6
	Walsh (2024)	Calculated	Distribution	9.6 10.8
KOOS ADL score	Qiao (2024)	Calculated	Distribution	10.0
	Retzky (2024)	Calculated	Distribution	10.2
	Walsh (2024)	Calculated	Anchor Distribution	7.2 9.4
KOOS Sports & Recreation score	Qiao (2024)	Calculated	Distribution	17.8
	Qiao (2024)	Established	Distribution	17.8
	Retzky (2024)	Calculated	Distribution	16.0
	Walsh (2024)	Calculated	Distribution	12.4 14.0
KOOS Quality of Life score	Qiao (2024)	Calculated	Distribution	12.7
	Qiao (2024)	Established	Distribution	12.7
	Retzky (2024)	Calculated	Distribution	13.2
	Walsh (2024)	Calculated	Distribution Anchor	12.2 12.4
KOOS JR Pain score	Walsh	Calculated	Distribution	7.5 7.9
KOOS JR Symptoms score	Walsh	Calculated	Distribution	7.6 8.1
KOOS JR ADL score	Walsh	Calculated	Distribution Anchor	7.7 25.2
VAS score	Bayoumi (2021)	Established	Distribution	20

*Note*: Qiao et al. = The value of minimal clinically important difference, substantial clinical benefit, and patient‐acceptable symptomatic state for commonly used patient‐reported outcomes in recurrent patellar instability patients after medial patellofemoral ligament reconstruction and tibial tubercle transfer. Qiao et al. = Double‐level knee derotational osteotomy yields better postoperative outcomes than tibial tubercle transfer combined with medial patellofemoral ligament reconstruction in patients with recurrent patellar instability and severe malrotation.

Abbreviations: ADLs, activities of daily living; BPI, Banff Patellar Instability Index; IKDC, International Knee Documentation Committee; JR, joint replacement; KOOS, Knee injury and Osteoarthritis Outcome Score; NR, not reported; VAS, visual analogue scale.

### SCB and PASS

Five studies (5/17; 29.4%) reported on SCB and PASS values consisting of 450 patients [24, 34, 39, 43, 50]. Two of the studies reported on SCB for 142 patients and calculated their values rather than using established ones [34, 50]. All five studies reported on PASS for 450 patients [24, 34, 39, 43, 50]. Three studies (3/5; 60%) calculated PASS values [34, 43, 50], while the other two studies (2/5; 40%) used established PASS values from those studies [24, 39]. Four studies (4/5; 80.0%) used anchor‐based methods to calculate SCB and PASS [24, 34, 39, 43], while one study used both anchor and distribution methods for their analysis [50].

A total of 14 different outcomes were given SCB and PASS values. For IKDC subjective scores, the SCB value ranged from 14.5 to 23.6, and the PASS value ranged from 64.9 to 83.3. For KOOS Pain, the SCB value ranged from 1.4 to 13.9, and the PASS value ranged from 76.8 to 87.5. For KOOS Symptoms, the SCB value ranged from 4.2 to 14.2, and the PASS values ranged from 73.2 to 83.3. For KOOS ADL, the SCB value ranged from 6.5 to 25.7, and the PASS value ranged from 91.2 to 99.3. For KOOS Sports and Recreation, the SCB values ranged from 47.5 to 55.0, and the PASS values ranged from 47.5 to 77.5. For KOOS Quality of Life, the SCB values ranged from 6.3 to 43.7, and the PASS values from 40.6 to 53.1. SCB and PASS values for Kujala, Kujala Function, Kujala Symptom, KOOS JR Pain, JOOS JR Symptom, KOOS JR ADL, Lysholm and Tegner Activity scores can be seen in Table 3.

**Table 3:** Substantial clinical benefit and patient acceptable symptom state values.

Patient‐reported outcome measure	Author	Calculated or established	Calculation	Substantial clinical benefit value	Patient acceptable symptom state value
IKDC score	Qiao (2024)	Calculated	Anchor	14.5	73.2
	Shi (2023)	Calculated	Anchor	NR	83.3
	Walsh	Calculated	Anchor	23.6	64.9 65.5
KOOS Symptom score	Long (2023)	Established	Anchor		80.4
	Qiao (2024)	Calculated	Anchor	14.3	73.2
	Walsh (2022)	Calculated	Distribution and Anchor	**14.3** 4.2	83.3 80.4
KOOS Pain score	Long (2023)	Established	Anchor		84.7
	Qiao (2024)	Calculated	Anchor	13.9	87.5
	Walsh (2022)	Calculated	Anchor	1.4 19.7	76.8 84.7
KOOS ADL score	Long (2023)	Established	Anchor		99.3
	Qiao (2024)	Calculated	Anchor	18.4	92.0
	Walsh (2022)	Calculated	Distribution and Anchor	**25.7** 6.5	91.2 99.3
KOOS Sport & Recreation score	Long (2023)	Established	Anchor		57.5
	Qiao (2024)	Calculated	Anchor	47.5	77.5
	Walsh (2022)	Calculated	Distribution and Anchor	**47.5** 55.0	47.5 57.5
KOOS QOL score	Long (2023)	Established	Anchor		53.1
	Qiao (2024)	Calculated	Anchor	15.0	53.1
	Walsh (2022)	Calculated	Anchor	43.7 6.3	40.6 53.1
Kujala Anterior Knee Pain Score	Runer (2024)	Established	Anchor		83.5
	Qiao (2024)	Calculated	Anchor	14.5	85.5
Kujala Function score	Walsh (2022)	Calculated	Distribution and Anchor	**30**	84.5 83.5
Kujala Symptom score	Walsh (2022)	Calculated	Distribution and Anchor	**13.5** **30.0**	NR
KOOS JR Pain score	Walsh (2022)	Calculated	Distribution and Anchor	**21.4** 19.6	78.1 76.3
KOOS JR Symptom score	Walsh (2022)	Calculated	Distribution and Anchor	**21.4** **27.1**	NR
KOOS JR ADL score	Walsh (2022)	Calculated	Distribution and Anchor	**23.5** 25.2	NR
Lysholm score	Qiao (2024)	Calculated	Anchor	12.5	75.5
Tegner Activity score	Qiao (2024)	Calculated	Anchor	1.5	3.5

*Note*: bold = distribution method.

Abbreviations: ADLs, activities of daily living; BPI, Banff Patellar Instability Index; IKDC, International Knee Documentation Committee; JR, joint replacement; KOOS, Knee Osteoarthritis and Outcome Score; NR, not reported; VAS, visual analogue scale.

### Patient achievement of CSOs

Twelve studies reported on percentages of their patient population achieving MCID, SCB or PASS thresholds [2, 7, 14, 24, 28, 32, 35, 39, 43, 51, 56, 57]. Out of five studies that reported SCB and PASS values, only two of them provided percentages of patients who achieved those values [39, 43]. Two studies used mean score differences between preoperative and postoperative statuses to evaluate improvement in MCID [34, 41].

For IKDC subjective scores, the patients that achieved MCID cutoffs ranged from 28% to 98.6%. For Kujala scores, 38%–100% of patients achieved MCID cutoffs. For Lysholm scores, 44%–98.4% of patients achieved MCID thresholds. For Tegner Activity, scores 84.0%–95% of 50%–89% of patients achieved cutoff MCID scores. For BPI 2.0 scores, 84.0%–95.0% of patients achieved MCID cutoffs. For KOOS Pain, 50%–83% of patients achieved cutoff MCID scores. For KOOS Sports and Recreation, 56%–89% of patients achieved cutoff MCID scores. For KOOS Quality of Life, 61%–94% of patients achieved MCID cutoffs (Table 4).

**Table 4:** Achievement of clinically significant outcomes.

Value	Outcome	Author	% Achieved
MCID	IKDC score	Hu (2024)	98.6 93.4
		Qiao (2024)	72 28
		Shi (2023)	95.2
	IKDC Function score	Waters (2024)	61.1 64.5
	KOOS score		78
	KOOS Pain score	Long (2023)	63
		Qiao (2024)	83 50
	KOOS Symptoms score	Long (2023)	71
	KOOS ADL score		38
	KOOS Sports and Recreation score	Long (2023)	67
		Qiao (2024)	89 56
	KOOS QOL score	Long (2023)	83
		Qiao (2024)	94 61
	Kujala Anterior Knee Pain Score	Bayoumi (2021)	84
		Hu (2024)	98.6 100
		Mao (2024)	94.1
		Qiao (2024)	38 50
	Lysholm score	Hu (2024)	97.2 98.4
		Qiao (2024)	78 44
	Tegner Activity score	Hu (2024)	77.8 72.1
		Mao (2024)	47.1
		Qiao (2024)	89 50
	BPI 2.0 score	Milinkovic (2023)	92.0 95.0
		Milinkovic (2024)	88.0
		Zimmerman (2020)	92.0 84.0
		Zimmerman (2023)	84.0 90.0
	VAS score	Bayoumi (2021)	78
PASS	IKDC score	Shi	66.7
	Kujala Anterior Knee Pain Score	Runer	79.7

Abbreviations: ADL, activities of daily living; BPI, Banff Patellar Instability Index; IKDC, International Knee Documentation Committee; JR, joint replacement; KOOS, Knee Osteoarthritis and Outcome Score; MCID, minimal clinically important difference; NR, not reported; PASS, patient acceptable symptom state; PROM, patient‐reported outcome measure; SCB, substantial clinical benefit; VAS, visual analogue scale.

## DISCUSSION

The primary finding of this systematic review was that MCID, PASS and SCB values generally had large variability in values between included studies that investigated surgical treatment for patellar instability. Values for MCID were calculated for just over half of all included studies (9/15, 60%), with the predominant technique being distribution‐based calculation. Furthermore, PASS and SCB were both far less discussed among studies compared to MCID, with only five studies reporting values, and around half of these studies using established values. Finally, the reported percentages of patients who achieved the thresholds for MCID and PASS of PROMs were all highly variable, potentially reflecting existing heterogeneity between these values in the literature.

There are several reasons that may exist for the heterogeneity reported in this review and that of others. Fourteen out of 15 studies reporting on MCID reported calculating MCID using a distribution‐based approach. This strategy calculates thresholds based on the standard error of the data, and the difference in values from before treatment to after [11]. However, small sample sizes tend to have larger variability, which can skew CSO values to be high [12]. The opposite effect would be observed in those with larger sample sizes [12]. The PASS value is calculated via an anchor‐based method which employs a more patient‐centred approach. For instance, each patient when filling out a questionnaire for a given PROM is asked if they are ‘satisfied’ with their treatment. ROC analyses are performed to distinguish the cutoff that separates those who are ‘satisfied’ and those who are not. The PASS is the AUC of the ROC. The MCID and SCB values are also calculated during follow‐up assessments by asking patients how much improvement they perceive in their condition. Little to moderate improvement would correspond with the MCID, and significant improvements would correspond with the SCB. ROC analyses would again be used to calculate the optimal cutoff points [12]. It has been recommended that studies utilize an anchor‐based approach as distribution‐based methods may not accurately result in what they are aiming to achieve [20].

This review included 12 of the 17 studies with fewer than 100 patients, raising concerns about their ability to calculate adequately sampled CSO values. A review article in 2022 with 101 patients assessed the effects of intrinsic variables on the calculation of MCID and found that changing the sample size and threshold for success affected MCID values differently depending on the PROM used [33]. Another systematic review in 2018 with 116 trials of hip and knee osteoarthritis found that core components of sample size calculations were only reported in 21%, with only about half of the studies having reproducible sample size calculations [6]. Given the various methodological differences in distribution and anchor MCID calculations, it is difficult to ascertain the effects of sample size and other variables on MCID score, especially if they are not shared in the literature. In many cases, the MCID is calculated a priori to establish sample sizes for larger clinical trials. However, the use of previous literature on established MCIDs or effect sizes should be used to estimate the required sample size.

Additional potential reasons for variability included surgical intervention, patient and demographic variables and follow‐up times [48]. However, the studies from this review may be less affected by these reasons, as almost all studies had patients undergo MPFLR and TTO. There is potential for patient variables to contribute towards variability, where patient baseline function is associated with changes in PROM scores [5]. For example, an older patient with greater disability would likely, from a subjective standpoint, report greater satisfaction and improvement compared to a young athlete undergoing the same procedure [5]. The reported patient ages were all within their 20s and the mean or median follow‐up time was over 24 months. Only one study deviated in age with a mean value of 16.1 years [28] and only one study deviated in follow‐up time, reporting a minimum of 6 months being necessary for patient inclusion [50].

Other issues pertaining to CSOs include using established values from pre‐existing literature and the lack of recognition of SCB and PASS. Six (40%) studies used established thresholds for MCID, two (40%) studies used established thresholds for PASS, while all two studies (100%) calculated values for SCB. One recent study reported on recommended guidelines regarding CSOs, recommending that ‘clinical importance should be uniquely determined for the studied patient population’ [12]. Differences in follow‐up times and demographics decrease the validity and the results when drawing comparisons using established values. The systematic review from 2021 reporting on CSOs for hip arthroscopy found similar rates of studies using established values to this review, with 55.1% of studies failing to calculate their own MCID values [20]. Without calculating MCID values, researchers risk errors in interpreting clinical relevance, as the applied MCID may not accurately reflect the specific population, timeline or intervention being studied. This can lead to inappropriate conclusions about the effectiveness of a treatment, and can overstate or understate its true clinical impact [4, 9, 52]. MCID has also been considered to be a ‘low bar’ to gauge clinical improvement, especially for patients who may be high‐level athletes [38]. Despite its cons, MCID's utility appears to be grounded in its applicability to determine minimal change rather than significant change, its use in study design, and more well‐established methodologies [38]. Instead, prioritizing SCB and PASS may be better in terms of evaluating who has gained significant benefit [38]. Despite this, SCB and PASS values were widely underreported for patellar stabilization surgery. Similar findings were found in the hip arthroscopy literature, with MCID being frequently reported more than that of SCB or PASS [48]. As with other sports medicine procedures, it is likely that patients who undergo patellar stabilization surgery aim for significant benefit as opposed to that of minimal benefit. It is unclear why established values are used so frequently and may reflect an increased soft‐ or hard‐ re requirements from journals to report on these metrics.

With an increasing incidence of patellar instability among paediatric and adolescent populations, this study can help clarify the usage of MCID, SCB and PASS in different clinical contexts [29]. Traditional statistical approaches for comparison analyses of PROMs can lead to misleading results, as statistical significance may not reflect clinical relevance [12], thus having a summary of thresholds and calculation methods for these metrics is essential. Outcome metrics such as MCID, PASS and SCB offer potential in measuring clinical significance to help the interpretation of studies investigating various interventions [12]. However, this review is in conjunction with several other reviews that suggest that there is heterogeneity in CSO thresholds for various studies. A recent review of 56 studies determined that substantial variability exists in the reporting and calculation of MCID, SCB and PASS for IKDC, KOOS, Tegner and Lysholm scores following arthroscopic ACL reconstruction, with SCB scores only being calculated for four studies [3]. Another review reported that MCID and PASS thresholds for PROMs following ACL reconstruction and meniscal repairs were highly susceptible to heterogeneity, even within the same sample as a result of changing the calculation method, with scores differing up to 30 points [27]. The same findings following surgical interventions on the hip have been observed, where one review reported considerable heterogeneity in MCID, SCB, and PASS values after hip arthroscopy for FAI, with MCID values differing up to 100% and SCB values up to 300% [48]. Another review from 2021 analyzed 59 articles related to hip arthroscopy, finding significant heterogeneity in MCID thresholds, partly due to using established MCID values that were not cohort‐specific in addition to broad follow‐up times [20]. The same conclusion was reached in another review of 41 studies investigating CSO thresholds for rotator cuff repair, despite limiting their threshold follow‐up time to a minimum of 12 months [22]. In other subspecialties of orthopaedic surgery, such as that of upper extremity, arthroplasty, and spine surgery, findings were identical suggesting this heterogeneity to be a fundamental issue in the way MCID, SCB and PASS are reported [16, 21, 53].

There are few limitations associated with this study. First, there were no Level I or II studies included in this review, with all studies being retrospective in nature. Because of this, the values reported in this review may be prone to bias due to the lower quality of data. Second, due to the nature of systematic reviews in general, there may have been some studies reporting on CSOs that may have been missed. However, three large and distinct databases were used, aiming to sufficiently address this possible limitation. Finally, as mentioned previously, there were limited reported values for SCB and PASS, while MCID values were predominantly calculated using distribution‐based techniques.

## CONCLUSION

The significant heterogeneity in reported thresholds for MCID, SCB and PASS across studies highlights critical challenges in interpreting results after patellar stabilization surgery, specifically regarding what constitutes a clinically relevant outcome. MCID was the most commonly reported metric and calculated predominantly using distribution‐based methods, with over half of the studies using previously established thresholds. PASS and SCB were widely underreported as well, suggesting a need for studies investigating patellar stabilization to prioritize the calculation of all three metrics, using anchor‐based techniques.

## AUTHOR CONTRIBUTIONS

Screening, extraction and writing: Ahmed Bilgasem. Writing and idea conception: Prushoth Vivekanantha. Writing and editing: Lauren Gyemi. Screening and extraction: Zackariyah Hassan. Writing and editing: David Slawaska‐Eng. Writing and editing: Amit Meena. Writing and editing: Shahbaz Malik. Writing, editing and supervision: Darren de SA.

## CONFLICT OF INTEREST STATEMENT

The authors declare no conflicts of interest.

## ETHICS STATEMENT

There are no relevant ethical disclosures pertaining to research involving human participants and/or animals, and informed consent was not necessary to develop this manuscript.

## Supporting information

Supporting information.

## Data Availability

Data may be made available upon reasonable request from prushoth.vivekanantha@medportal.ca.
