# Supplementary material for: Large variability in minimal clinically important difference, substantial clinical benefit and patient acceptable symptom state values among literature investigating patellar stabilization surgery: A systematic review
Source: Knee Surg Sports Traumatol Arthrosc. 2025 May 6;34(2):445–58. doi: 10.1002/ksa.12684 (PMC12850594; doi:10.1002/ksa.12684)
Supplement: Supplementary file 1 — Supporting information. [file KSA-34-445-s001.docx]

**SUPPLEMENTARY DIGITAL MATERIAL:**

**Table 1.** Search Criteria

| **PubMed (n=722)** | **EMBASE (n=39)** | **MEDLINE[Ovid] (n=26)** |
| --- | --- | --- |
| 1) patellar dislocation OR patellofemoral instability OR patellar instability OR  patellar dislocation OR patellofemoral dislocation OR medial patellofemoral  ligament OR MPFL OR tibial tubercle osteotomy OR TTO  2) MCID OR minimal clinically important difference OR PASS OR patient  acceptable symptom state OR SCB OR substantial clinical benefit OR CSO OR  clinically significant outcome) | 1) patellar dislocation OR patellofemoral instability OR patellar instability OR  patellar dislocation OR patellofemoral dislocation OR medial patellofemoral  ligament OR MPFL OR tibial tubercle osteotomy OR TTO  2) MCID OR minimal clinically important difference OR PASS OR patient  acceptable symptom state OR SCB OR substantial clinical benefit OR CSO OR  clinically significant outcome) | 1) patellar dislocation OR patellofemoral instability OR patellar instability OR  patellar dislocation OR patellofemoral dislocation OR medial patellofemoral  ligament OR MPFL OR tibial tubercle osteotomy OR TTO  2) MCID OR minimal clinically important difference OR PASS OR patient  acceptable symptom state OR SCB OR substantial clinical benefit OR CSO OR  clinically significant outcome) |
| 3) 1 AND 2 | 3) 1 AND 2 | 3) 1 AND 2 |
